# HealthyPlan.City: A Web Tool to Support Urban Environmental Equity and Public Health in Canadian Communities

**DOI:** 10.1007/s11524-024-00855-x

**Published:** 2024-04-08

**Authors:** Dany Doiron, Eleanor M. Setton, Joey Syer, Andre Redivo, Allan McKee, Mohammad Noaeen, Priya Patel, Gillian L. Booth, Michael Brauer, Daniel Fuller, Yan Kestens, Laura C. Rosella, Dave Stieb, Paul J. Villeneuve, Jeffrey R. Brook

**Affiliations:** 1 Respiratory Epidemiology and Clinical Research Unit, Research Institute of the McGill University Health Centre, Montréal, Québec Canada; 2 Geography Department, University of Victoria, Victoria, BC Canada; 3 Dalla Lana School of Public Health, University of Toronto, Toronto, ON Canada; 4 Department of Medicine, University of Toronto, Toronto, ON Canada; 5 School of Population and Public Health, The University of British Columbia, Vancouver, BC Canada; 6 Department of Community Health and Epidemiology, College of Medicine, University of Saskatchewan, Saskatoon, SK Canada; 7 École de Santé Publique de L’Université de Montréal, Montréal, QC Canada; 8 Environmental Health Science and Research Bureau, Health Canada, Vancouver, BC Canada; 9 Department of Neuroscience, Carleton University, Ottawa, ON Canada

**Keywords:** Urban health, Environmental equity, Web mapping, Vulnerable populations, Environmental exposures, Urban planning

## Abstract

Urban environmental factors such as air quality, heat islands, and access to greenspaces and community amenities impact public health. Some vulnerable populations such as low-income groups, children, older adults, new immigrants, and visible minorities live in areas with fewer beneficial conditions, and therefore, face greater health risks. Planning and advocating for equitable healthy urban environments requires systematic analysis of reliable spatial data to identify where vulnerable populations intersect with positive or negative urban/environmental characteristics. To facilitate this effort in Canada, we developed HealthyPlan.City (https://healthyplan.city/), a freely available web mapping platform for users to visualize the spatial patterns of built environment indicators, vulnerable populations, and environmental inequity within over 125 Canadian cities. This tool helps users identify areas within Canadian cities where relatively higher proportions of vulnerable populations experience lower than average levels of beneficial environmental conditions, which we refer to as *Equity priority areas*. Using nationally standardized environmental data from satellite imagery and other large geospatial databases and demographic data from the Canadian Census, HealthyPlan.City provides a block-by-block snapshot of environmental inequities in Canadian cities. The tool aims to support urban planners, public health professionals, policy makers, and community organizers to identify neighborhoods where targeted investments and improvements to the local environment would simultaneously help communities address environmental inequities, promote public health, and adapt to climate change. In this paper, we report on the key considerations that informed our approach to developing this tool and describe the current web-based application.

**Supplementary Information:**

The online version contains supplementary material available at 10.1007/s11524-024-00855-x.

## Introduction

Chronic disease morbidity and mortality are linked with urban environmental exposures and attributes including air quality [1], neighborhood greenness [2], urban heat islands [3], healthy food availability [4], and the extent to which individuals can walk and cycle to their destinations (i.e., active transportation) [5]. Lower socioeconomic status (SES) neighborhoods are correlated with less beneficial environmental conditions such as higher air pollution concentrations and summer temperatures, and lower neighborhood greenness, walkability, and active transportation opportunities, which in turn negatively affect health outcomes [6–10].

Equitable healthy urban (re)development requires a systematic analysis of reliable spatial data to identify where vulnerable populations intersect with positive or negative urban/environmental characteristics. Technically, this can be accomplished easily given the current state of web-enabled mapping applications, the existence of comprehensive national small-area demographic data, and increasingly available neighborhood-level environmental data, at least in high-income countries around the world [11]. In Canada, some large cities have undertaken data-driven environmental equity initiatives. The *Equity Bold Move* program in the City of Vancouver combines geographic data on park provision, recreational opportunities, and access to nature to help identify areas with uneven service and resource distribution in order to prioritize investments and improve equity [12]. Neighborhood equity indices built off the World Health Organization’s Urban HEART (Urban Health Equity Assessment and Response Tool) framework [13] and accompanying maps were developed in Toronto [14], Ottawa [15], and Calgary [16] to identify the most vulnerable neighborhoods in each city, set priorities and actions to reduce social and health inequities across all neighborhoods, and monitor changes over time. In 2023 the City of Montreal implemented the Living Environment Equity Index, a territorial index designed to locate areas with a combination of urban vulnerabilities in order to prioritize and focus municipal investments [17]. However, smaller cities and communities often lack the capacity to produce these kinds of tools, conduct the analyses required, and/or disseminate knowledge. In addition, the lack of standardization in the methods and data used to generate indicators across existing tools leads to limited between-city comparability and substantial duplication of effort. This represents an important gap in the national surveillance of built environment determinants of health, and especially limits our understanding of how various impacts of the built environment are distributed among different population groups (i.e., low-income groups, children, older adults, new immigrants, and visible minorities).

To address this gap, we developed HealthyPlan.City, a web-enabled mapping application using nationally standardized neighborhood level data for 129 municipalities across Canada, with an explicit built environment equity focus. Herein, we report on the key considerations that informed our approach and describe the current web-based application.

## Methods

We developed a freely available web mapping platform that allows users to visualize the spatial patterns of built environment indicators, vulnerable populations, and environmental inequity within Canadian cities. In developing this application, we further aimed (i) to create a flexible data system for easy updating of datasets, and (ii) to engage users and obtain continuous feedback during the development process.

We identified target user groups for HealthyPlan.City as professional urban planners, environmental health practitioners, and community and advocacy groups. An Advisory Committee was recruited at the beginning of the design phase via a call for volunteers through the Canadian Urban Environmental Health Research Consortium (CANUE), the National Collaborating Centre for Environmental Health, and the Canadian Institute of Planners. We selected 16 individuals representing local governments, not-for-profit organizations, academia, and private industry from across Canada, as well as two internationally recognized experts on healthy cities. These individuals provided guidance on priority indicators and useful tool functions via meetings and surveys.

We collaborated with the University of Toronto’s Masters of Information (MI) program to test and improve the HealthyPlan.City tool user interface. Student teams worked on early versions as part of their course requirements for the MI User Experience Design concentration and subsequently were hired to develop and implement a comprehensive user experience evaluation program. Overall, 38 individuals representing urban planners (*n* = 15), public health professionals (*n* = 8), researchers (*n* = 7), community advocates (*n* = 5), policy makers (*n* = 2), and other (*n* = 1) took part in a comprehensive real-time testing interview of HealthyPlan.City prior to the last phase of development. Ease of use and clarity of content were the key foci of this effort.

We worked with a web development company to produce a custom web application that leverages a collection of geographic boundaries and a database of demographic and environmental data. During the development phase, we identified administrative functions to be built by the application developers that allow (i) easily updating the database without any coding necessary; (ii) editing all the text and images in the application interface; and (iii) gathering data for evaluation of uptake and usage.

### Geographic Boundary Files

The current implementation of HealthyPlan.City includes 129 municipalities (also called census subdivisions by Statistics Canada and commonly recognized as cities). We selected all census subdivisions (CSDs) with a population over 30,000 that intersected with one or more of Statistics Canada’s medium and large population centers (PCs), defined as areas with a population of at least 30,000 and a population density of 400 persons or more per square kilometer (see Supplemental Fig. S1). Since some CSD boundaries cover a large geographic area with a dispersed population, we excluded CSDs where less than 75% of the population live within the overlapping PCs. These population level and density thresholds help ensure that there is enough range in data to support the calculations required by the tool and that the tool includes data for the majority of individuals living in a given municipality. To facilitate the comparison of equity indicators among similar sized cities, we grouped the cities into five categories based on population: 30,000 to 50,000; 50,000 to 100,000; 100,000 to 200,000; 200,000 to 500,000; and over 500,000 residents. Within each CSD (city), we used census dissemination blocks (DBs), the smallest possible geographic zones available with total population counts reported, as the base geography, and assigned all demographic and environmental data to these DBs. For some cities, and if publicly available, we included boundaries such as planning areas, electoral districts, and locally defined neighborhoods to allow comparisons between subzones of a given city. We acquired the neighborhood boundaries from municipal open data portals.

### Demographic Data

We defined vulnerable populations as groups of individuals who, due to greater exposure, greater susceptibility to harm, and/or lower ability to prepare for or cope with hazards, may experience greater health impacts from environmental stressors and built environment factors relative to the general population. Specific vulnerable populations were identified based on existing literature and include visible minorities, low-income individuals, immigrants, children, older adults, and individuals living alone [18]. While individuals with pre-existing health conditions can also be considered vulnerable populations, such data is not available for small area geographic units and were therefore not included in the tool (Supplemental Table S1). The demographic indicators included in the current version of HealthyPlan.City were obtained from the 2021 Canadian Census as the percentage of a given vulnerable population within dissemination areas (DAs), which typically have total populations of 400 to 700 individuals. Within each city, we downscaled the DA-level data to the smaller dissemination block (DB) level by assigning the same proportion of vulnerable populations within a DA to all DBs within it, and for some summary reporting, we used the total population of a DB and the DA percentage to calculate the total number of people in a selected demographic group at the DB level.

### Built Environment Data

Built environment indicators were developed by the project data specialists for four themes identified as priorities by the Advisory Committee and Research Team—urban climate, community amenities, parks and recreation, and air and noise pollution. To the extent possible the data selected for the indicators needed to have complete coverage across Canada. Within each theme, the project data specialists explored available data sources for suggested indicators, and developed methods to produce and then assign the indicator values to DBs. For variables such tree canopy cover, we averaged all values of a 30-m raster within each DB of participating cities. To calculate the accessibility of different community amenities such as parks, retail, and services and transit stops, we summed the number of urban features within a 1-km buffer distance from DB centroids. This distance was chosen as it approximates the number of amenities in a short (10–15 min) walk from each DB [19]. We used a pragmatic approach during this phase that focused on developing indicators based on available data within a theme, rather than defining a specific indicator and then trying to find supporting data. The environmental data included in the current version of HealthyPlan.City are listed in Supplemental Table S1. For further details, and as both demographic and environmental indicators might change over time, please refer to the “Methods” section of the HealthyPlan.City website for the most up-to-date information (https://healthyplan.city/en/methods).

### Equity Priority Areas

**Fig. 1:**
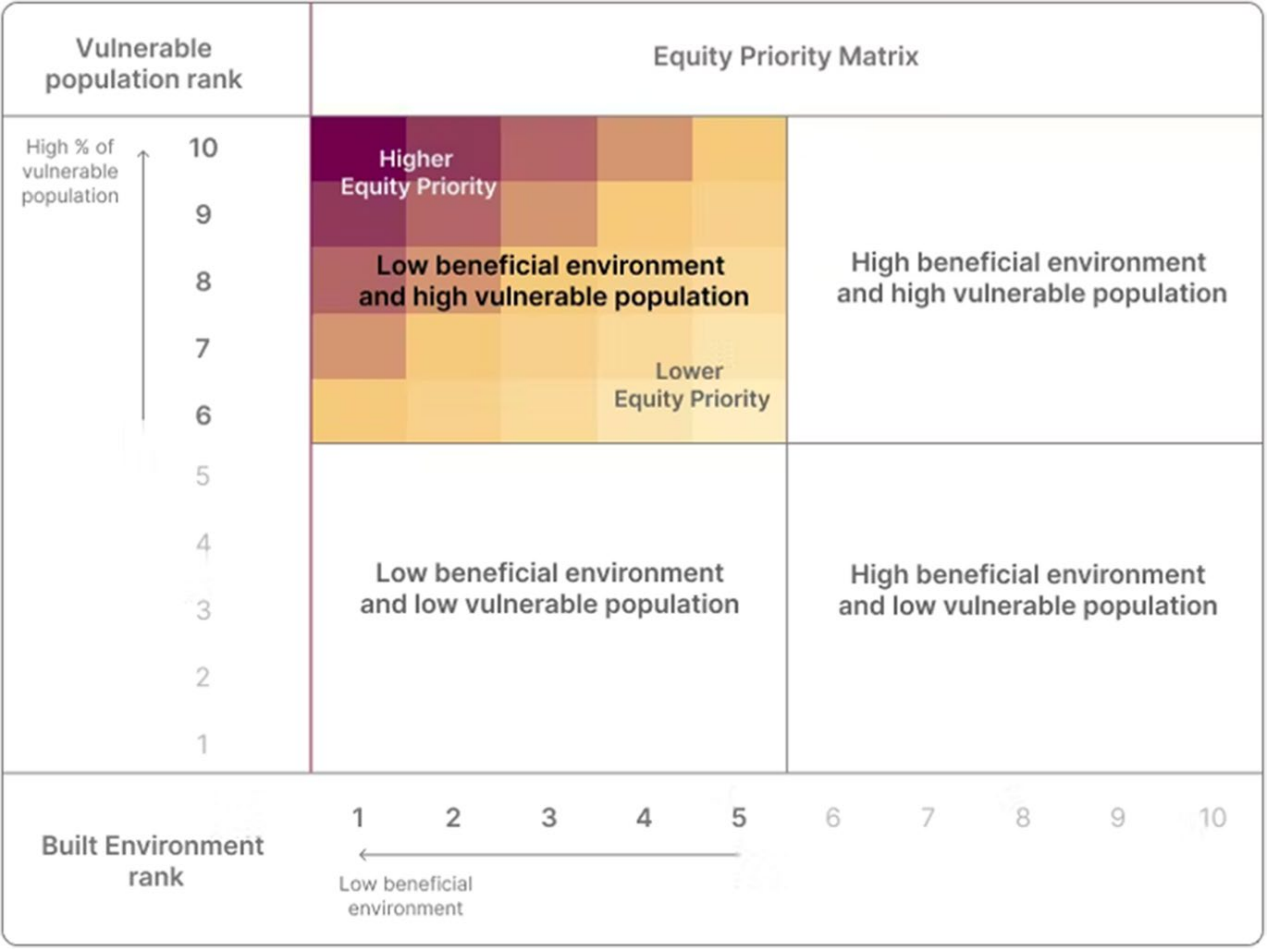
Equity priority matrix

By combining the demographic data with the environmental data, we identified *Equity priority* *areas* as dissemination blocks where proportionally more vulnerable people experience proportionally less beneficial environments. We first ranked the percentages of vulnerable populations and the values of built environment indicators for all populated dissemination blocks into city-specific deciles (i.e., from 1 to 10). A ranking of 1 represents the lowest 10% of values in a given city (i.e., low percentage of vulnerable population group or the lowest levels of beneficial environment), while a ranking of 10 represents the highest 10% of values (i.e., highest percentage of vulnerable population group or the highest levels of beneficial environment). Based on this ranking strategy, equity priority areas are identified by the intersection of city-level ranks of vulnerable populations > 5 (higher percentages of vulnerable populations) and built environment < 6 (lower levels of beneficial environments), as illustrated in the equity priority matrix in Fig. 1, and displayed accordingly in the map view. Darker shades of red show areas with higher inequity (values that are further from the city median for both indicators), whereas lighter shades of yellow show areas with lower inequity (closer to the city median).

The final data product is a comma separated value (csv) file with geographic, census, environmental, and equity priority values for every dissemination block within each city included in HealthyPlan.City. Geographic data columns include the dissemination block unique identifier, the unique municipality name to which each dissemination block belongs, a city group variable identifying similar-sized cities across Canada, and the unique neighborhood zone names within select cities. Census data columns include the population counts and the percent values of each vulnerable population for each dissemination block. The environmental data columns include values for built environment indicators (e.g., # of parks within a 1-km radius from the DB, average percent coverage of tree canopy cover within a DB). Finally, two columns indicating the city-specific decile ranks of demographic and environmental variables are included to allow identifying equity priority areas.

### Administrative and Research Data Functions

The platform was designed to enable indicators to change whenever new, better, or simply different data are identified or become available. Doing so requires changes to the associated database; therefore, the application developers created a stand-alone script that allows the project team to update or replace specific datasets on the PostgreSQL database with new versions as needed. In addition to the database and geographic files, a substantial amount of content (text, images, links, tutorials, etc.) was required to help users navigate and understand the maps and summary data views presented in HealthyPlan.City. A comprehensive content management system (CMS) using Prismic.io was established to allow easy editing of this type of information. Currently, Google Analytics is enabled and will allow for tracking site use to support research on how HealthyPlan.City is being used. Importantly, these flexible attributes enable us to adapt the application for targeted uses. For example, we can copy the application and database to a new unique domain/url and include a specific geographic zone(s) and/or data of interest. This functionality could be useful for a city with specific local data or key issues on which they want to focus, or for a broader application on a specific topic.

## Results

Based on multiple iterations with advisors and feedback from early testers, the final HealthyPlan.City application includes three key user interface sections: the left hand panel, the map view, and the data visualization window. The left hand panel offers users a tutorial, dropdown menus to select options for the map view, and access to supporting information, including examples of environmental equity initiatives in Canadian communities, a mailing list signup, a PDF report of the selected city and indicators, details on HealthyPlan.City methods and data, information on the research team and funding, and a data request form.

**Fig. 2:**
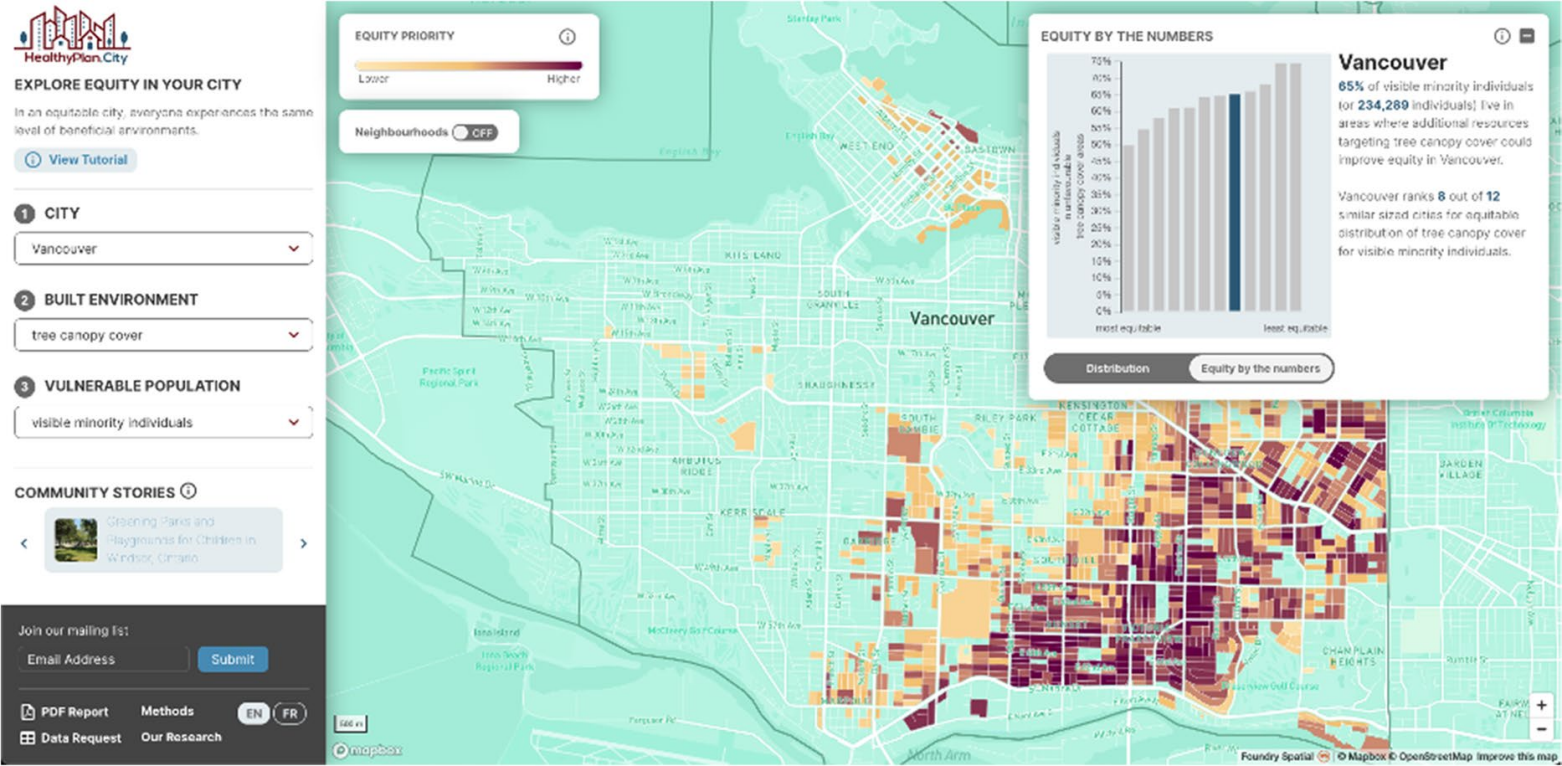
The HealthyPlan.City interface showing *Equity priority areas*: tree canopy cover and visible minority individuals in the City of Vancouver

The map view responds to the selections made in the left hand panel. Any time a city (dropdown 1), built environment (dropdown 2), and vulnerable population (dropdown 3) are selected from the dropdown menus, an equity priority map is produced. The equity priority map allows easy identification of areas where environmental inequity is present within each city in the HealthyPlan.City tool. For example, the map shown in Fig. 2 outlines areas in the City of Vancouver where higher proportions of visible minorities and relatively lower tree canopy cover coincide. As mentioned above, deeper hues denote higher percentiles of vulnerable populations and less beneficial environments, and could be considered higher equity priorities for that city.

**Fig. 3:**
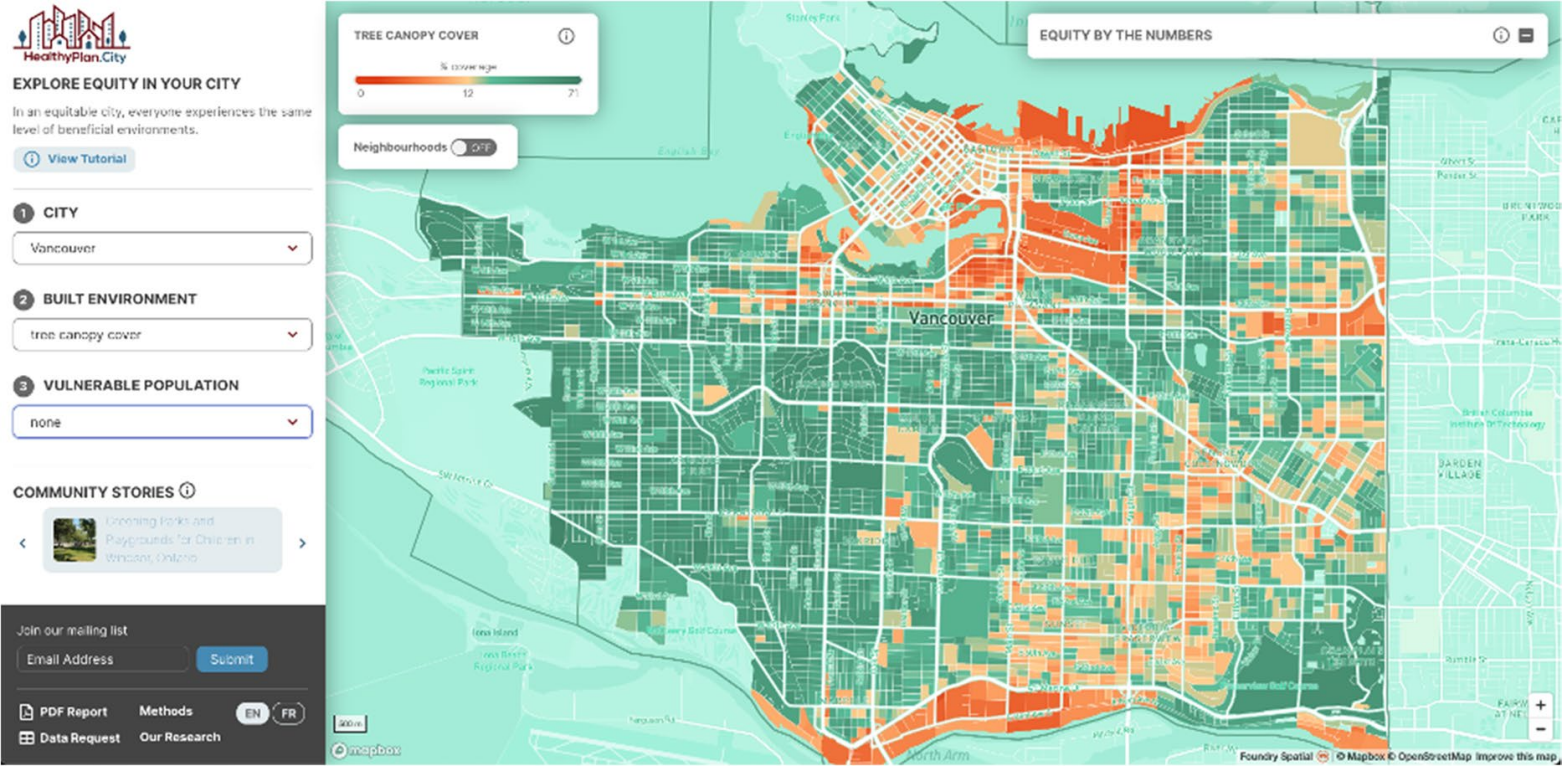
The HealthyPlan.City interface showing the distribution of a single indicator: tree canopy cover in the City of Vancouver

If the user selects “none” for one of dropdowns 2 or 3, the map will display only the distribution of either the selected built environment indicator or the vulnerable population for the selected city. Built environment maps illustrate how conditions vary across the selected city, with the legend appearing in the top left. In the example shown in Fig. 3, selecting only the tree canopy cover layer under the built environment dropdown menu displays the spatial distribution of this variable for the City of Vancouver. Similarly, vulnerable population maps illustrate the proportion of the selected population in each DB. Users can zoom in, pan, and click on DBs to get a pop-up with the data values.

**Fig. 4:**
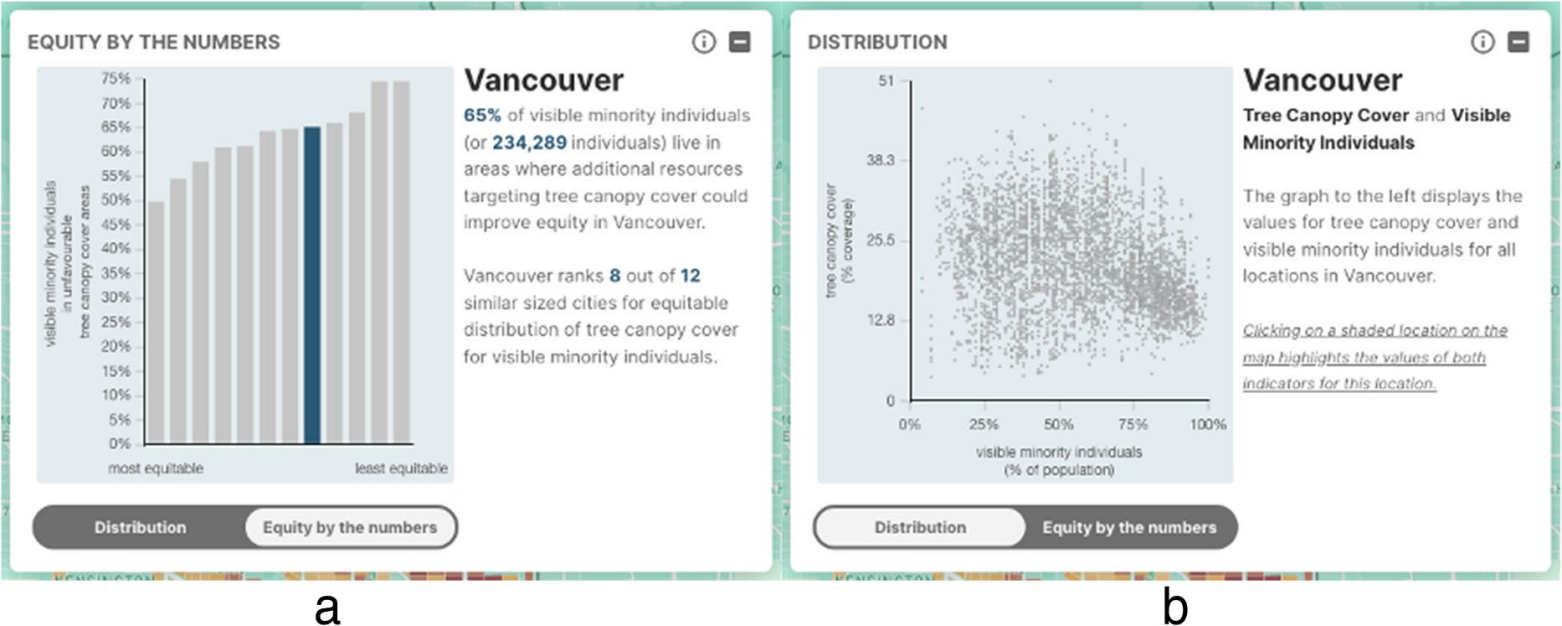
**a**, **b** The *Equity by the numbers* and *Distribution* data visualization screens: tree canopy cover and visible minority individuals in the City of Vancouver

The data visualization window appears (Fig. 2 top right) only when the user makes a built environment and a vulnerable population selection, and presents two “screens,” accessible by toggling between the *Equity by the numbers* and the *Distribution* tabs at the bottom of the window. The *Equity by the numbers* screen provides the city-wide proportion and number of individuals from vulnerable populations living in less beneficial environments for the selected indicators. This screen also provides a histogram that compares the proportion of the selected vulnerable population living in less beneficial environments in the selected city to that of similar-sized cities. Hovering over columns displays the names of the other cities shown. For example, Fig. 4a shows the proportion (and number) of visible minority individuals living in areas with relatively low tree canopy cover in Vancouver and ranks this city in relation to other cities with over 500,000 residents.

The *Distribution* screen shows a plot of built environment indicator values against vulnerable population percentages for all DBs in the selected city. Although only DBs with inequity are displayed in the map, we include data for all DBs in the plot in order to show the full range of data for the selected city. Clicking on a colored DB in the map highlights the associated data point in the plot. Figure 4b shows an example of this function where the values of tree canopy cover percentage and proportion of visible minority individuals for all DBs in the City of Vancouver are plotted against each other.

**Fig. 5:**
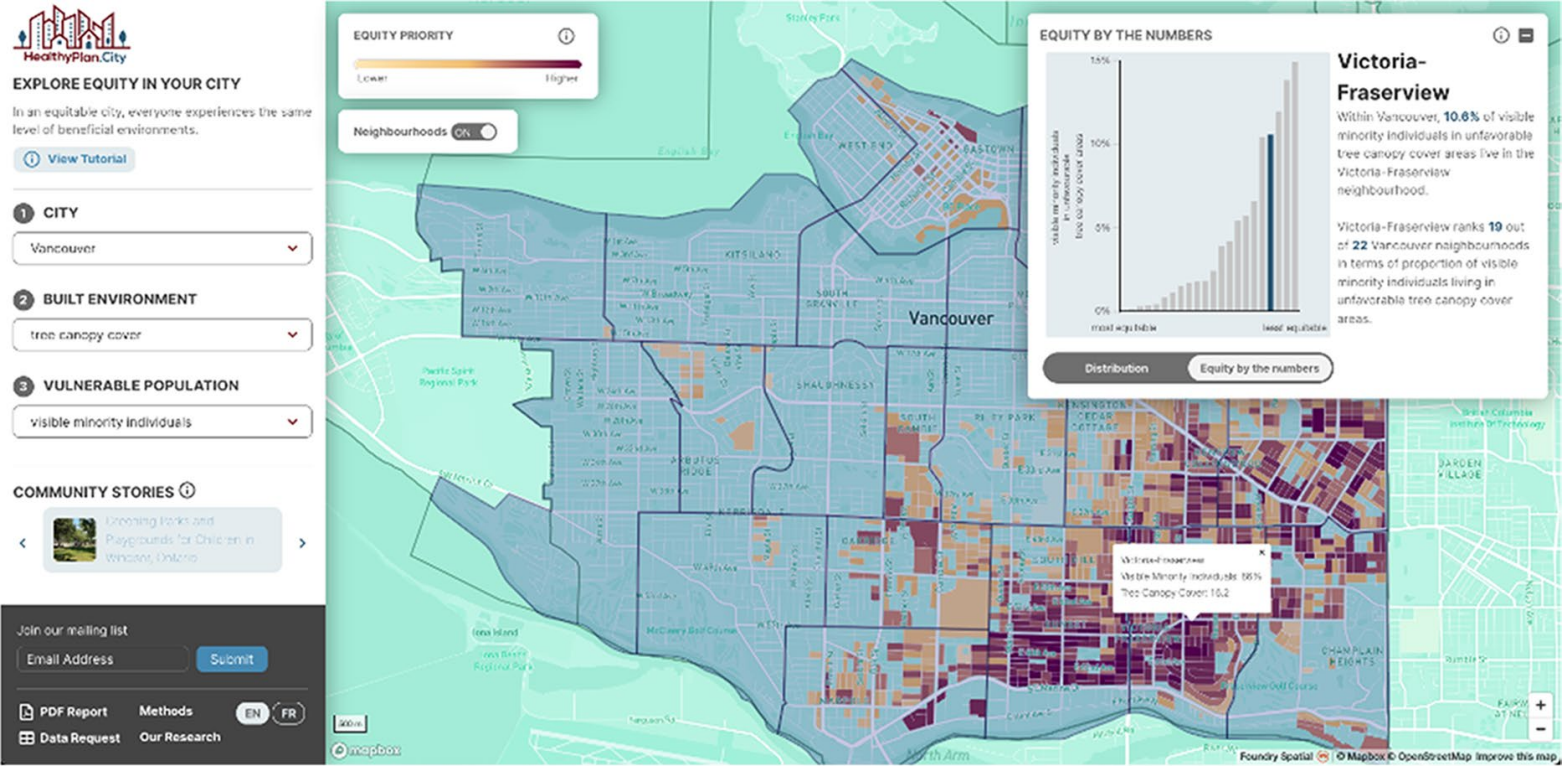
The Healthy Plan. City interface showing neighborhood equity rankings: tree canopy cover and visible minority individuals in the City of Vancouver

When neighborhood zones are available for a selected city, the “Neighbourhoods on/off” toggle appears and users have access to summary data to compare neighborhoods within the selected city. When Neighbourhoods is toggled on, their boundaries appear on the map and the *Equity by the numbers* screen presents the neighborhood-specific percentage of the city-wide selected vulnerable population living in equity priority dissemination blocks. This screen also compares all neighborhoods within the selected city with each other. Figure 5 shows an example of the Neighbourhood function for the City of Vancouver. The map displays the boundaries for all 22 Vancouver neighborhoods, while the *Equity by the numbers* screen ranks them in terms of equitable distribution of tree canopy cover for visible minority individuals. Toggling to the *Distribution* screen shows a plot of aggregated values for all dissemination blocks within each neighborhood zone and displays each zone as a dot in a scatter plot, enabling easy comparison across neighborhoods in a given city.

## Discussion

HealthyPlan.City is a web-based tool that facilitates assessment of environmental inequity as it relates to built environment indicators and vulnerable populations in Canadian cities. This custom-built tool integrates nationally standardized data on a variety of health-relevant and in some cases climate change–relevant built environment indicators and demographic data from the Canadian Census. Users can map the spatial patterns of built environment indicators, vulnerable populations, and environmental inequity within cities. Functions within the tool also allow for comparisons of environmental inequity across different cities and across neighborhoods of a selected city. Throughout the development process, the team leading the HealthyPlan.City initiative relied on expertise and feedback from individuals in local governments, not-for-profit organizations, academia, and private industry to ensure its relevance for key stakeholders. HealthyPlan.City was developed as a flexible and scalable tool; it allows easily adding or modifying underlying built environment and demographic indicators or geographic boundaries to update it over time and/or meet the needs of specific types of users.

### Urban Health Indicator and Equity Tools

The exponential growth in computing power, software, and digital data has led to a proliferation in the number of urban indicator initiatives to measure progress on improving the well-being of urban residents. Indeed, it is difficult to overstate how many unique indicators have been proposed that characterize urban living and therefore how challenging it can be to select a manageable set of indicators that are readily interpretable for users for any particular initiative. A recent review of 145 urban health indicator (UHI) tools from around the world found they contained 8006 different indicators covering more than 20 domains, ranging from air quality to education, food environment, transportation, and leisure and culture, among others [20]. The myriad of possible indicators leads to significant difficulty in comparing conclusions derived from different equity-related products among jurisdictions such as between countries and even within countries.

Additional indicator complexity can be introduced when multiple indicators are combined into a single summary index and/or when a weighting scheme is applied to indicators [21]. For example, equity indices developed by the cities of Toronto, Ottawa, and Calgary combine numerous normalized indicators using principal component analysis to assign weights prior to summing into an index that is meant to illustrate overall inequity [14–16]. The Environmental Justice Index (EJI) Explorer tool [22] combines 36 indicators across social, health, and environmental domains using percentile rankings to produce a single index value for all census tracts in the USA so any census tract can be compared with any other via mapping. Along with the overall index, the EJI Explorer provides details on each of the included indicators in pop-up windows. Similarly, the US Environmental Protection Agency’s Environmental Justice Screening and Mapping Tool (EJ Screen) calculates an Environmental Justice Index as the intersection between percent low-income and percent people of color with one of 13 different environmental indicators. Finally, the Tree Equity Score tool [23] is another example of a cumulative disadvantage index approach that combines data on tree canopy cover, climate, demographic, and socioeconomic status to identify areas in US cities with greater priority for tree planting.

The methods used to measure inequities in and among urban areas using spatial data vary widely across initiatives. Some tools have made use of numerical thresholds (or benchmarks) to identify disadvantaged communities as an illustration of inequity. For example, the Climate and Economic Justice Screening (CEJS) Tool [24] contains US census tract-level data (converted to percentiles) on 29 indicators across eight domains—climate change, energy, health, housing, legacy pollution, transportation, waste and wastewater, and workforce development. A community is classified as disadvantaged if at least one of the indicators in a domain is at the 90th percentile or higher and one of two vulnerable population indicators (proportion of low income population or those with high school education) is above the 65th percentile. While a data window provides the total community population and ethnic makeup in the CEJS Tool, there is no easy quantification of the magnitude of exceedance (i.e., how many indicators are exceeded).

HealthyPlan.City includes some features in common with the approaches described above and has the advantage of offering a standard approach for all the selected Canadian cities. The ability for users to explore the actual distribution of built environment and vulnerable population indicators and, in parallel, map *Equity priority areas* based on above/below median normalized values for both indicators allow users to appreciate the distribution of inequity in a city. The color scale used for *Equity priority areas* provides users with clear mapping of where both indicators are further from the median. Dissemination block level population counts also allow us to derive the percentages and numbers of people affected. In practice, our demographic indicators (i.e., children, older adults, and low income individuals) are analogous to the social and economic indicators included in the tools described above, and our environmental indicators are similar as well, although they focus more narrowly on health-relevant urban built environment indicators.

### Limitations

Local knowledge is key to evaluating and prioritizing potential policy change or targeted programs to improve environmental conditions for vulnerable populations. As discussed previously, the equity priority rank in HealthyPlan.City is based on normalizing both environmental and demographic data into deciles and using the median (50th) percentile as a threshold to identify equity priority areas. Users should keep in mind that the range of raw values for a given indicator varies considerably between cities and is generally larger for bigger centers relative to smaller communities. For example, the proportion of low-income visible minority individuals within dissemination blocks in the city of Toronto (population = 2,794,356) ranges from 0 to 36%, while this same indicator ranges from 0 to 5% in Langford, BC (population = 46,584). Likewise, ambient nitrogen dioxide air pollution concentrations range from 5.9 to 15.2 parts per billion (ppb) in Toronto and 3.3 to 7.4 ppb in Langford. Although *Equity priority areas* will be highlighted for each of these cities in HealthyPlan.City, the absolute difference between *Equity priority areas* and non-shaded areas in Toronto will be much larger than in Langford.

Downscaling of demographic data from DAs to smaller DBs also represents a limitation in that the proportion of vulnerable populations at the DA level may not be the same in each of the underlying DBs. This however is mainly a problem in less densely populated areas where DAs are composed of many adjacent DBs. We decided to downscale demographic data to take advantage of high-resolution spatial data that captures the variation of urban environmental conditions in equity analyses.

Some environmental data might also be incomplete, not updated, or simply contain errors or anomalies. For example, we use data extracted from Open Street Map (OSM) data to produce indicators on proximity measures such as retail and services, healthy food outlets, and large natural spaces. While OSM is relatively complete in urban areas of Canada, data are crowd sourced and coding of features can therefore be inconsistent. Nonetheless, OSM remains one of the few free data sources for location data in Canada. Indicators that incorporate satellite data can be affected by changes in satellite sensors over time, overpass frequency and time of day, and the presence of clouds, all which could affect the output data and may be difficult to compensate for over time. The flexibility of the tool’s architecture will allow relatively easy inclusion of new or improved data as it becomes available.

### Early Applications of HealthyPlan.City

The first official version of HealthyPlan.City was released in November, 2023. We previously deployed a limited version of the tool focusing on urban heat in conjunction with media stories published by the Canadian Broadcasting Corporation about health and heatwaves [25–27]. Early versions of HealthyPlan.City also supported work done by Green Communities Canada (GCC), by supporting 11 community organizations across Canada in planning for and implementing equitable green infrastructure projects. These organizations made use of information provided by HealthyPlan.City to help ensure that green infrastructure projects are situated in areas with high environmental and social needs. Specifically, HealthyPlan.City provided equity maps for each participating community that depict environmental risk factors (e.g., places with higher-than-average surface temperatures, higher-than-average flood risk, and lower-than-average tree canopy cover) relative to social risk factors (e.g., neighborhoods with higher proportions of low-income and visible minority individuals) to help prioritize areas within each municipality for equity-oriented greening interventions such as tree planting, rain gardens, and depaving.

## Conclusion

HealthyPlan.City is an evolving tool. Our aim is to disseminate it to as many interested stakeholders as possible and gather suggestions and feedback to improve its usability and functionalities, to integrate appropriate indicators or data sources, and ensure that it serves its purpose to simultaneously help communities address environmental inequities, promote public health, and adapt to climate change.

### Supplementary Information

ESM1 DOCX (745 KB)

## Data Availability

Built environment data included in the HealthyPlan.City tool is available for non-commercial purposes. More information is available in the Data Request section of the tool at https://healthyplan.city/en.
