# Supplementary material for: HealthyPlan.City: A Web Tool to Support Urban Environmental Equity and Public Health in Canadian Communities
Source: J Urban Health. 2024 Apr 8;101(3):497–507. doi: 10.1007/s11524-024-00855-x (PMC11190122; doi:10.1007/s11524-024-00855-x)
Supplement: Supplementary file 1 — DOCX (745 KB) [file 11524_2024_855_MOESM1_ESM.docx]

**Supplemental information**

**HealthyPlan.City: A Web Tool to Support Urban Environmental Equity and Public Health in Canadian Communities**

Dany Doiron^1^*^†^, Eleanor M Setton^2^*, Joey Syer^3^, Andre Redivo^2^, Allan McKee^3^, Mohammad Noaeen^3^, Priya Patel^3^, Gillian L Booth^4^, Michael Brauer^5^, Daniel Fuller^6^, Yan Kestens^7^, Laura C Rosella^3^, Dave Stieb^8^, Paul Villeneuve^9^, Jeffrey R Brook^3^

^1^ Respiratory Epidemiology and Clinical Research Unit, Research Institute of the McGill University Health Centre, Montréal, Québec, Canada

^2^ Geography Department, University of Victoria, Victoria, BC, Canada

^3^ Dalla Lana School of Public Health, University of Toronto, Toronto, ON, Canada

^4^ Department of Medicine, University of Toronto, Toronto, ON, Canada

^5^ School of Population and Public Health, The University of British Columbia, Vancouver BC Canada

^6^ Department of Community Health and Epidemiology, College of Medicine, University of Saskatchewan.

^7^ École de santé publique de l'Université de Montréal, Canada, QC, Canada

^8^ Environmental Health Science and Research Bureau, Health Canada, Vancouver, BC, Canada

^9^ Department of Neuroscience, Carleton University, Ottawa, ON, Canada

*Co-first authors

^†^ Correspondence to:

D. Doiron, Respiratory Epidemiology and Clinical Research Unit,  Research Institute of the McGill University Health Centre, email: dany.doiron@mail.mcgill.ca

**Figure S1:** The municipal (CSD) boundary for the City of Regina is shown in blue. All areas in green have a population density of 400 person/km2 and represent the Population Centres (PC) for which data are mapped within municipal boundaries.


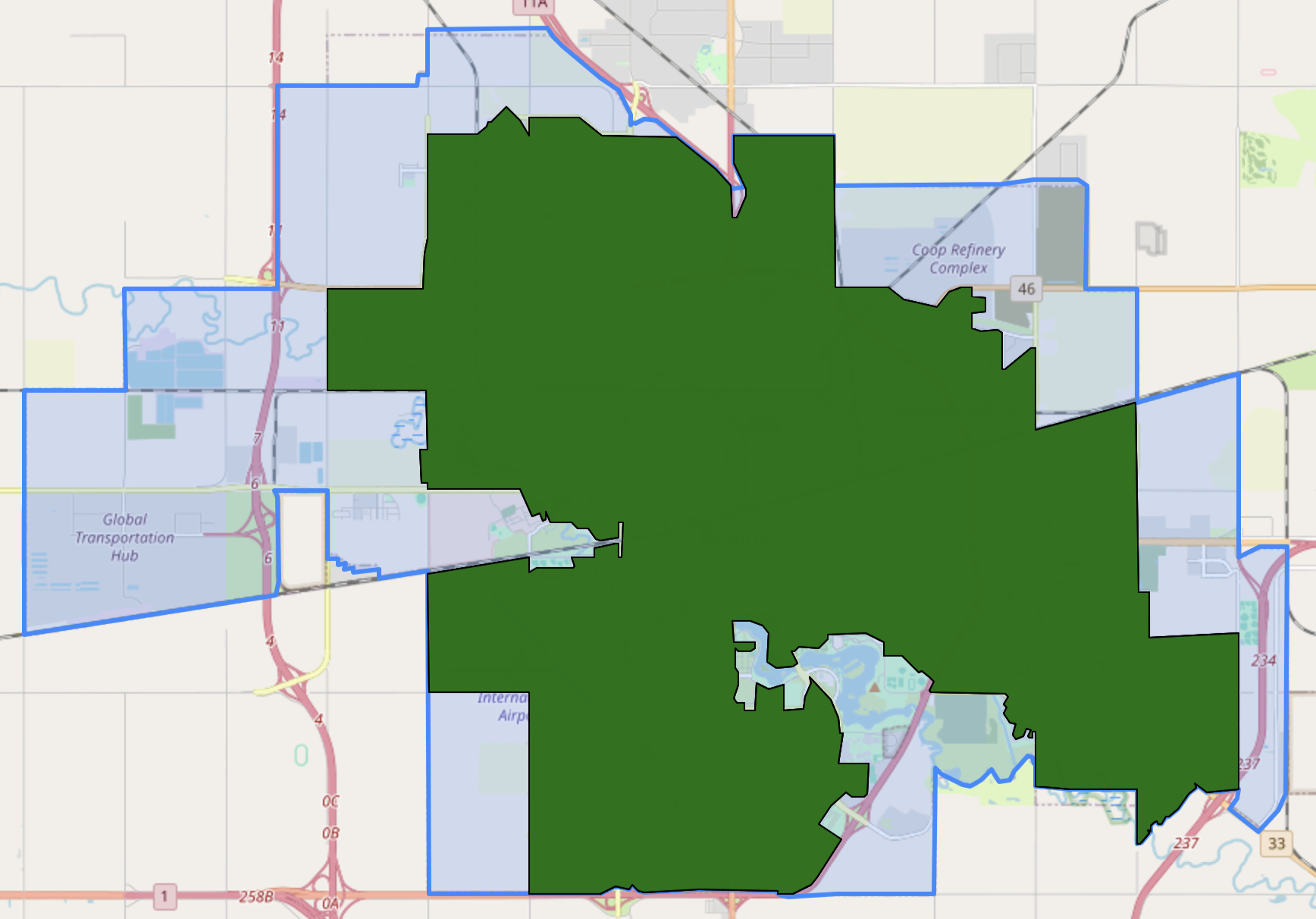


**Table S1:** Demographic and environmental data

|  | **Variable and definition** | **Source** |
| --- | --- | --- |
| **Demographic indicators** | **Children:** *Percentage of population under 15 years of age* | **2021 Canadian Census** |
|  | **Older adults:** *Percentage of population over 64 years of age* |  |
|  | **Visible minority individuals:** *Percentage of population that identify as a visible minority* |  |
|  | **Individuals living alone**: *Percentage of population in private households who live alone* |  |
|  | **Newly-arrived immigrants**: *Percentage of population who are immigrants to Canada between 2011 and 2021* |  |
|  | **First-generation immigrants**: *Percentage of population who are immigrants born outside of Canada* |  |
|  | **Low-income individuals**: *Percentage of population under the after-tax low-income threshold* |  |
|  | **Low-income children**: *Percentage of children (under 18 y/o) under the after-tax low-income threshold* |  |
|  | **Low-income older adults***: Percentage of older adults (over 64 y/o) under the after-tax low-income threshold* |  |
|  | **Low-income visible minority individuals**: *Percentage of population under the after-tax low-income cut-off threshold that identify as a visible minority* |  |
|  | **Low-income individuals living alone**: *Percentage of population under the after-tax low-income cut-off threshold that live alone* |  |
|  | **Low-income newly-arrived immigrants**: *Percentage of population under the after-tax low-income cut-off threshold who are immigrants to Canada between 2011 and 2021* |  |
|  | **Low-income first-generation immigrants**: *Percentage of population under the after-tax low-income cut-off threshold who are immigrants born outside of Canada* |  |
| **Built environment indicators** | **Average summer temperature:** *Mean land surface temperature from May to September 2019-2021, averaged within dissemination blocks.* | **LandSat 8 satellite imagery** |
|  | **Tree canopy cover:** *Satellite-derived vegetation coverage from trees and large woody plants, averaged within dissemination blocks.* | **LandSat 8 satellite imagery[1]** |
|  | **Flood susceptibility:** *Areas prone to flooding based on patterns of historic flood events, averaged within dissemination blocks.* | **Natural Resources Canada's Flood Susceptibility Index[2]** |
|  | **Transit stops:** *Number of public transit amenities such as bus and tram stops, subway stations and car sharing locations within a 1 km radius from dissemination block centroids.* | **Open Database of Infrastructure[3]** |
|  | **Education and culture:** *Number of facilities such as schools, libraries, museums, and event venues within a 1 km radius from dissemination block centroids.* | **OpenStreetMap database, extracted in November 2022** |
|  | **Retail and services:** *Number of outlets such as stores, restaurants, professional services, and pharmacies within a 1 km radius from dissemination block centroids.* | **OpenStreetMap database, extracted in November 2022** |
|  | **Healthy food outlets:** *Number of fresh food outlets such as supermarkets, groceries, delis, and markets within a 1 km radius from dissemination block centroids.* | **OpenStreetMap database, extracted in November 2022** |
|  | **Large natural areas:** *Number of forests, beaches, wetlands and nature reserves within a 1 km radius from dissemination block centroids.* | **OpenStreetMap database, extracted in November 2022** |
|  | **Parks:** *Number of community gardens, neighbourhood parks and large parks within a 1 km radius from dissemination block centroids.* | **OpenStreetMap database, extracted in November 2022** |
|  | **Recreation and sports facilities:** *Number of facilities such as arenas, swimming pools, fitness centers and bowling alleys within a 1 km radius from dissemination block centroids.* | **OpenStreetMap database, extracted in November 2022** |
|  | **Air pollution:** *Nitrogen dioxide air pollution for the year 2016, averaged within dissemination blocks.* | **Developed by Hystad et al.[4]** |
|  | **Noise pollution:** *Number of noise pollution features within a distance that varies by their type (ranging from 250 m to 1 km), including airports, runways, helipads, railroads, fire and ambulance stations, and major roads.* | **OpenStreetMap database, extracted in November 2022** |

1. Sexton, J.O., et al., *Global, 30-m resolution continuous fields of tree cover: Landsat-based rescaling of MODIS vegetation continuous fields with lidar-based estimates of error.* International Journal of Digital Earth, 2013. **6**(5): p. 427-448.

2. McGrath, H. and P.N. Gohl, *Prediction and Classification of Flood Susceptibility Based on Historic Record in a Large, Diverse, and Data Sparse Country.* Environmental Sciences Proceedings, 2023. **25**(1): p. 18.

3. Statistics Canada. *The Open Database of Infrastructure*. 2023 [cited 2023 Dec 14]; Available from: <https://www150.statcan.gc.ca/n1/pub/34-26-0003/342600032023001-eng.htm>.

4. Hystad, P., et al., *Creating national air pollution models for population exposure assessment in Canada.* Environmental Health Perspectives, 2011. **119**(8): p. 1123-1129.
